# Supplementary figures and images for: Metabolite profiling, antifungal, biofilm formation prevention and disruption of mature biofilm activities of Erythrina senegalensis stem bark extract against Candida albicans and Candida glabrata
Source: PLoS One. 2022 Nov 28;17(11):e0278096. doi: 10.1371/journal.pone.0278096 (PMC9704668; doi:10.1371/journal.pone.0278096)

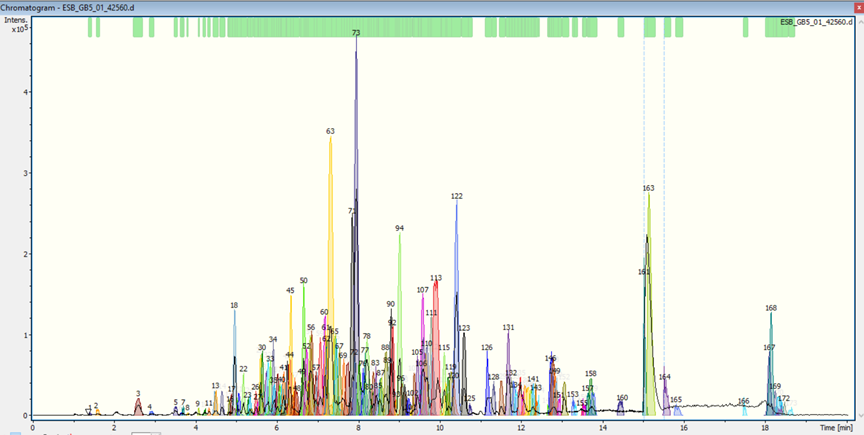

Supplement: S1 Fig — (TIF) [file pone.0278096.s001.tif]
