# Supplementary material for: Metabolite profiling, antifungal, biofilm formation prevention and disruption of mature biofilm activities of Erythrina senegalensis stem bark extract against Candida albicans and Candida glabrata
Source: PLoS One. 2022 Nov 28;17(11):e0278096. doi: 10.1371/journal.pone.0278096 (PMC9704668; doi:10.1371/journal.pone.0278096)
Supplement: S1 File — (DOCX) [file pone.0278096.s002.docx]

**S1 Table: The inhibitory effect of ESB and standard antifungals fluconazole and capsofungin against resistant *C. albicans* clinical isolates**

| Clinical isolate/Sample | Minimum Inhibitory Concentration (µg/mL) | | |
| --- | --- | --- | --- |
|  | **ESB** | **Fluconazole** | **Caspofungin** |
| CA-1 (azole-R) | 16 | >128 | 4 |
| CA-2 (azole-R) | 16 | >128 | 4 |
| CA-3 (azole-R) | 8 | >128 | 4 |
| CA-4 (azole-R) | 8 | 128 | 4 |
| CA-5 (azole-R) | 16 | >128 | 8 |
| CA-6 (nystatin-R) | 8 | 128 | 8 |
| CA-7 (nystatin-R) | 16 | 128 | 4 |
| CA-8 (nystatin-R) | 64 | >128 | 8 |

ESB: *E. senegalensis* 70% ethanol stem bark extract, CA: Clinical isolate of *C. albicans*, azole-R: azole-resistant isolate, nystatin-R: nystatin-resistant isolate

**S2 Table: The inhibitory effect of ESB and standard antifungals fluconazole and capsofungin against resistant *C. glabrata* clinical isolates**

| Clinical isolate/Sample | Minimum Inhibitory Concentration (µg/mL) | | |
| --- | --- | --- | --- |
|  | **ESB** | **Fluconazole** | **Caspofungin** |
| CG-1 (azole-R) | 4 | 64 | 2 |
| CG-2 (azole-R) | 4 | 64 | 2 |
| CG-3 (azole-R) | 4 | 64 | 2 |
| CG-4 (azole-R) | 8 | 64 | 2 |
| CG-5 (azole-R) | 4 | 64 | 4 |
| CG-6 (nystatin-R) | 8 | 64 | 2 |
| CG-7 (nystatin-R) | 8 | 64 | 4 |
| CG-8 (nystatin-R) | 16 | 64 | 2 |

ESB: *E. senegalensis* 70% ethanol stem bark extract, CG: Clinical isolate of *C. glabrata*, azole-R: azole-resistant isolate, nystatin-R: nystatin-resistant isolate

**S3 Table: Compounds identified in the leaves of *E. senegalensis* from UPLC-ESI-QTOF-MS/MS analysis**

| S/N | Retention time (min) | | Observed m/z [Adduct(s)] | Monoisotopic mass | MS/MS Fragments | Accuracy (ppm/mDa) | Molecular Formula | Identification | Reference |
| --- | --- | --- | --- | --- | --- | --- | --- | --- | --- |
| 1 | 4.08 | 318.14 (M+NH_4_) | | 300.10 |  | 8 mDa |  | Eryvarin H | 1, 11 |
| 2 | 4.24 | 462.15 (M+H) | | 461.14 | - | 20 ppm | C_24_H_31_NO_8_ | Glucoerysodine | 2 |
| 3 | 4.48 | 301.11 (M+H) | | 300.11 | 119, 167 | 4 mDa | C_16_H_12_O_6_ | Erythbidin D | 1 |
| 4 | 4.48 | 371.23 (M+H) | | 370.22 | - | 4 mDa | C_20_H_18_O_7_ | 4-O-methylsigmoidin B | 12 |
| 5 | 4.66 | 609.84 (M+H) | | 608.18 | 463, 191 | 5 ppm | C_31_H_28_O_13_ | epicatechin-(4beta->8)-4'-O-methylgallocatechin |  |
| 6 | 4.83 | 481.26 (M+Na) | | 458.28 | - | 20 ppm | C_30_H_50_O_3_ | Soyasapogenol B | 1 |
| 7 | 4.83 | 565.16 (M+H) | | 564.15 | - | 10 ppm | C_26_H_28_O_14_ | Schaftoside | 1 |
| 8 | 4.86 | 617.19 (M+Na) | | 594.20 | - | 20 ppm | C_27_H_30_O_15_ | Vicenin-2 | 1,4 |
| 9 | 5.03 | 321.10 (M+Na) | | 298.11 | - | 8 mDa | C_17_H_14_O_5_ | Erysubin C | 1 |
|  | 5.30 | 265.13 (M+Na) | | 242.14 | - | 8 mDa | C_14_H_10_O_4_ | 2-(2,4-dihydroxyphenyl)-6-hydroxybenzofuran | 13 |
| 10 | 5.53 | 393.10 (M+H) | | 392.09 | - | 8 mDa | C_25_H_28_O_4_ | Erybraedin A | 1, 2 |
| 11 | 6.07 | 415.17 (M+Na) | | 392.18 | - | 20 ppm | C_25_H_28_O_4_ | Erybraedin C | 1, 2 |
| 12 | 6.17 | 255.07 (M+H) | | 254.24 | - | 2 mDa | C_15_H_10_O_4_ | 3,9-Dihydroxypterocarp-6a-en | 1 |
| 13 | 6.39 | 353.10 (M+H) | | 352.10 | 283, 169, 121 | 4 mDa | C_21_H_20_O_5_ | Sigmoidin H | 1 |
| 14 | 6.45 | 337.24 (M+H) | | 336.23 | - | 4 mDa | C_20_H_16_O_5_ | Alpinumisoflavone | 1, 5 |
| 15 | 7.07 | 453.13 (M+H) | | 452.15 | - | 5 ppm | C_26_H_28_O_7_ | Erysenegalensein C | 6 |
| 16 | 7.19 | 355.12 (M+H) | | 354.11 | - | 2 mDa | C_21_H_22_O_5_ | Cristacarpin | 1 |
| 17 | 7.32 | 439.17 (M+H) | | 438.17 | - | 5 ppm | C_25_H_26_O_7_ | Erysenegalensein D | 1, 5, 7, 8 |
| 18 | 7.64 | 439.17 (M+H) | | 438.17 | - | 5 ppm | C_25_H_26_O_7_ | Erysenegalensein O | 1, 5, 7, 8, 11 |
| 19 | 8.85 | 423.18 (M+H) | | 422.17 | - | 5 ppm | C_25_H_26_O_6_ | 2,3-Dihydroauriculatin | 1, 9 |
| 20 | 8.74 | 369.13 (M+H) | | 368.13 | - | 2 mDa | C_22_H_24_O_5_ | Erythribyssin A | 1 |
| 21 | 8.94 | 439.18 (M+H) | | 438.17 | - | 20 ppm | C_25_H_26_O_7_ | Erysenegalensein N | 1, 5, 7, 8, 11 |
| 22 | 9.02 | 371.15 (M+H) | | 370.14 | - | 2 mDa | C_21_H_22_O_6_ | Lysisteisoflavone | 11 |
| 23 | 9.07 | 369.17 (M+H) | | 368.16 | - | 4 mDa | C_22_H_24_O_5_ | Erythribyssin C | 1, 11 |
| 24 | 9.14 | 369.17 (M+H) | | 368.17 | - | 4 mDa | C_22_H_24_O_5_ | Erythribyssin E | 1, 11 |
| 25 | 9.21 | 437.16 (M+H) | | 436.16 | - | 10 ppm | C_25_H_24_O_7_ | Erysenegalensein F | 1, 11 |
| 26 | 9.24 | 424.19 (M+NH_4_) | | 406.15 | - | 20 ppm | C_26_H_30_O_4_ | Erycristin | 1, 11 |
| 27 | 9.38 | 423.18 (M+H) | | 422.17 | - | 10 ppm | C_26_H_30_O_5_ | Abyssinone V 4'-O-Methyl Ether | 1, 11 |
| 28 | 9.59 | 337.11 (M+H) | | 336.10 | - | 2 mDa | C_20_H_16_O_5_ | Derrone | 1, 5, 8, 10, 11 |
| 29 | 9.69 | 421.80 (M+H) | | 420.79 | - | 5 ppm | C_25_H_24_O_6_ | Auriculatin | 1, 5, 8, 10, 11 |
| 30 | 9.83 | 355.15 (M+H) | | 354.15 | - | 2 mDa | C_21_H_22_O_5_ | Cristacarpin | 1, 11 |
| 31 | 10.21 | 423.18 (M+H) | | 422.17 | - | 10 ppm | C_25_H_26_O_6_ | 8-prenylluteone | 11 |
| 32 | 10.42 | 407.19 (M+H) | | 406.18 | - | 5 ppm | C_25_H_26_O_5_ | Erylysin A | 1, 11 |
| 33 | 10.60 | 407.19 (M+H) | | 406.18 | - | 5 ppm | C_25_H_26_O_5_ | Erythrisenegalone | 1, 2, 11 |
| 34 | 11.52 | 363.25 (M+Na) | | 340.26 | 317, 262, 195, 81 | 2 mDa | C_20_H_20_O_5_ | Dolichin isomer | 1, 11 |
| 35 | 11.69 | 405.17 (M+H) | | 404.17 | - | 5 ppm | C_25_H_24_O_5_ | Scandenone | 1, 11 |
| 36 | 11.76 | 349.11 (M+H) | | 348.11 | - | 8 mDa | C_22_H_20_O_4_ | Erybraedin E | 1, 11, |
| 37 | 11.98 | 345.24 (M+Na) | | 322.25 | 277, 179 | 2 mDa | C_20_H_18_O_4_ | Neobavaisoflavone | 1, 2, 11 |
| 38 | 12.18 | 429.28 (M+Na) | | 406.29 | - | 10 ppm | C_25_H_26_O_5_ | 6,8-diprenylgenistein | 1, 2, 5, 8, 10 |

**References**

1. Son NT, Elshamy AI. Flavonoids and other Non-alkaloidal Constituents of Genus *Erythrina*: Phytochemical Review. Combinatorial Chemistry & High Throughput Screening 2021; 24(1): 20-58. <https://dx.doi.org/10.2174/1386207323666200609141517>
2. Fofana S, Ouédraogo M, Esposito RC, Ouedraogo WP, Delporte C, Van Antwerpen P, Mathieu V, Guissou IP. Systematic Review of Potential Anticancerous Activities of *Erythrina senegalensis DC (Fabaceae)*. Plants. 2022; 11(1):19. <https://doi.org/10.3390/plants11010019>
3. Cui L, Thuong PT, Lee HS, Ndinteh DT, Mbafor JT, Fomum ZT, Oh WK. Flavanones from the stem bark of *Erythrina abyssinica*. Bioorganic & Medicinal Chemistry. 2008; 16(24): 10356-10362. <https://doi.org/10.1016/j.bmc.2008.10.012>
4. Pérez AJ, Hassan EM, Pecio L, Omer EA, Kucinska M, Murias M, Stochmal A. Triterpenoid saponins and C-glycosyl flavones from stem bark of *Erythrina abyssinica* Lam and their cytotoxic effects. Phytochemistry Letters. 2015; 13: 59-67. <https://doi.org/10.1016/j.phytol.2015.05.013>
5. Lee JS, Oh WK, Ahn JS, Kim YH, Mbafor JT, Wandji J, Fomum ZT. Prenylisoflavonoids from Erythrina senegalensis as novel HIV-1 protease inhibitors. Planta Medica. 2009; 75(3): 268-270. https://doi.org/10.1055/s-0028-1088395.
6. Thomas N, Zachariah SM. Pharmacological activities of chromene derivatives: an overview. Asian Journal of Pharmaceutical and Clinical Research. 2013; 6(Suppl 2): 11-15.
7. Bilanda DC, Dzeufiet PDD, Fouda YB, Ngapout RF, Tcheutchoua Y, Owona PE, Wouamba SCN, Tatchou LT, Dimo T, Kamtchouing P. Antihypertensive and antidiabetic activities of Erythrina senegalensis DC (Fabaceae) stem bark aqueous extract on diabetic hypertensive rats, Journal of Ethnopharmacology. 2020; 246: 112200. https://doi.org/10.1016/j.jep.2019.112200.
8. Oh WK, Lee C-H, Seo JH, Chung MY, Cui L, Fomum ZT, Kang JS, Lee HS. Diacylglycerol acyltransferase-inhibitory compounds from Erythrina senegalensis. Archives of Pharmacal Research. 2009; 32: 43-47. https://doi.org/10.1007/s12272-009-1116-2.
9. Taylor RB, Corley DG, Tempesta MS, Fomum ZT, Ayafor JF, Wandji J, Ifeadike PN. 2,3-Dihydroauriculatin, a new prenylated isoflavanone from *Erythrina senegalensis*. Application of the selective INEPT technique. Journal of Natural Products. 1986; 49(4): 670–673. <https://doi.org/10.1021/np50046a019>
10. Oh WK, Lee HS, Ahn SC, Ahn JS, Mbafor JT, Wandji J, Fomum ZT, Chang HK, Kim YH. Prenylated isoflavonoids from Erythrina senegalensis. Phytochemistry. 1999; 51(8): 1147-1150. https://doi.org/10.1016/S0031-9422(99)00171-5.
11. Fahmy NM, Al-Sayed E, El-Shazly M, Singab AN. Comprehensive review on flavonoids biological activities of Erythrina plant species. Industrial Crops and Products. 2018; 123: 500-538. https://doi.org/10.1016/j.indcrop.2018.06.028.
12. Koch K, Schulz G, Döring W, Büchter C, Havermann S, Mutiso PC, Passreiter C, Wätjen W. Abyssinone V, a prenylated flavonoid isolated from the stem bark of Erythrina melanacantha increases oxidative stress and decreases stress resistance in Caenorhabditis elegans. Journal of Pharmacy and Pharmacology. 2019; 71(6): 1007–1016. <https://doi.org/10.1111/jphp.13074>
13. Tanaka H, Hattori H, Sato M, Yamaguchi R, Fukai T, Tanaka T, Sakai E. New constituents from the roots of *Erythrina x bidwillii*. Heterocycles. 2007; 71(8): 1779-1785. J-GLOBAL ID: 200902209370831775 Reference number: 07A0716548
