# Supplementary material for: Metabolite profiling, antifungal, biofilm formation prevention and disruption of mature biofilm activities of Erythrina senegalensis stem bark extract against Candida albicans and Candida glabrata
Source: PLoS One. 2022 Nov 28;17(11):e0278096. doi: 10.1371/journal.pone.0278096 (PMC9704668; doi:10.1371/journal.pone.0278096)
Supplement: Attachment — Submitted filename: Review 27 06 22.docx [file pone.0278096.s003.docx]

Dear Editor-In-Chief

Plos one

Greetings…!

Upon careful revision on the submitted research article shortly entitled “Short title: Antifungal activities of the stem bark extract of *Erythrina senegalensis* against *Candida albicans and Candida glabrata-*PONE-D-22-14963 - [EMID:5055810f925a7bde]*”* the following points are suggested to enhance the standard of this research.

The following comments needs to be addressed before acceptance:

Recommended to add the reference for the zone inhibition line 145-150

Age of the fungal suspension used for “2.6.1 Inhibition of biofilm formation assay line 199”

Justify why the authors used solvent 70% ethanol, why not different concentrations and other solvents.

There are many references available the phenolic compounds such as flavonoids and alkaloids from plant sources has the ability to inhibit the fungal pathogens especially yeasts. The authors need to suggest the novelty of this research by highlighting a potential antifungal compound (S) based on UPLC fingerprints or any new compound(s) which are not reported before.

The write-up flow of the article is scientifically well described and easy to follow. The methods used by the authors are up to date.

Further the authors are recommended to read and cite the article to substantiate this research.

Al Aboody, M. S., & Mickymaray, S. (2020). Anti-fungal efficacy and mechanisms of flavonoids. *Antibiotics,* *9*, 45. [**https://doi.org/10.3390/antibiotics9020045**](https://doi.org/10.3390/antibiotics9020045)

Mickymaray, S.; Alturaiki, W. (2018). Antifungal efficacy of marine macroalgae against fungal isolates from bronchial asthmatic cases. Molecules, **23**, 3032. [**https://doi.org/10.3390/molecules23113032**](https://doi.org/10.3390/molecules23113032).

<https://doi.org/10.1016/j.apjtb.2015.12.005>

Best regards
