# Supplementary material for: Metabolite profiling, antifungal, biofilm formation prevention and disruption of mature biofilm activities of Erythrina senegalensis stem bark extract against Candida albicans and Candida glabrata
Source: PLoS One. 2022 Nov 28;17(11):e0278096. doi: 10.1371/journal.pone.0278096 (PMC9704668; doi:10.1371/journal.pone.0278096)
Supplement: Attachment — Submitted filename: Response to reviewers comments.docx [file pone.0278096.s005.docx]

The Editor-In-Chief

PLOS ONE

Cambridge, UK

Dear Madam,

**RESPONSE TO REVIEWERS’ COMMENTS**

We hereby in this letter provide responses to the queries raised by the academic editor and reviewers of our revised manuscript titled “Metabolite profiling, antifungal, biofilm formation prevention and disruption of mature biofilm activities of Erythrina senegalensis stem bark extract against Candida albicans and Candida glabrata” submitted to your revered journal PLOS One.

Kindly find below our responses.

We hope it will receive a favourable consideration.

Thank you.

Yours faithfully,

**Benjamin Kingsley Harley (*Ph.D*)**

**(Corresponding Author)**

**Journal Requirements:**

**Please review your reference list to ensure that it is complete and correct. If you have cited papers that have been retracted, please include the rationale for doing so in the manuscript text or remove these references and replace them with relevant current references. Any changes to the reference list should be mentioned in the rebuttal letter that accompanies your revised manuscript. If you need to cite a retracted article, indicate the article’s retracted status in the References list and also include a citation and full reference for the retraction notice.**

The reference list has been reviewed and it is complete and correct. It has also been formatted to meet the journal requirements.

**Additional Editor Comments:**

**According to journal style, the highlight must be removed from the manuscript. follow the journal style for text, tables and figures**

All highlights have been removed from the manuscript. We have ensured to adhere to the journal style for text, tables and figures.

**Reviewer 3**

**1. The study presents the results of original research.**

**2. Results reported have not been published elsewhere.**

**3. Experiments, statistics, and other analyses are performed to a high technical standard and are described insufficient detail.**

**4. Conclusions are presented in an appropriate fashion and are supported by the data.**

**5. The article is presented in an intelligible fashion and is written in standard English.**

**6. The research meets all applicable standards for the ethics of experimentation and research integrity.**

**7. The article adheres to appropriate reporting guidelines and community standards for data availability.**

The authors express outmost gratitude for the review and comments submitted.
